# Proposing Bromo-Epi-Androsterone (BEA) for Stiff Person Syndrome (SPS)

**DOI:** 10.3390/microorganisms13040824

**Published:** 2025-04-05

**Authors:** Coad Thomas Dow

**Affiliations:** 1 McPherson Eye Research Institute, University of Wisconsin-Madison, Madison, WI 53705, USA; ctdow@wisc.edu; 2 Protibea Therapeutics, LLC., Naples, FL 34105, USA

**Keywords:** stiff person syndrome (SPS), glutamic acid decarboxylase (GAD), *Mycobacterium* avium *subspecies* (MAP), molecular mimicry, type 1 diabetes (T1D), Dehydroepiandrosterone (DHEA), Bromo-Epi-Androsterone (BEA)

## Abstract

SPS is characterized by progressive spasmodic muscular rigidity. SPS is thought to be an autoimmune disease with a prominent feature of antibodies against glutamic acid decarboxylase (GAD). GAD is responsible for the enzymatic conversion of glutamic acid (glutamate) into the inhibitory neurotransmitter gamma-aminobutyric acid (GABA). Reduced GABA activity leads to increased excitability in the central nervous system, resulting in muscle rigidity and spasms characteristic of SPS. While SPS is rare, anti-GAD antibodies seen in SPS are also seen in the much more common autoimmune disease, type 1 diabetes (T1D). There is evolving research showing that the anti-GAD antibodies of T1D are produced in response to the presence of mycobacterial heat shock protein 65 (mHSP65), and the mHSP65 is produced in response to an occult infection by a bacterium, *Mycobacterium* avium *subspecies Paratuberculosis* (MAP). Humans are broadly exposed to MAP in food, water, and air. There are linear and conformational similarities between the epitopes of GAD and mHSP65. This article proposes that MAP is also an infectious trigger for SPS. Dehydroepiandrosterone (DHEA) is a principal component of the steroid metabolome; it plateaus in young adults and then steadily declines. Bromo-epi-androsterone (BEA) is a potent synthetic analog of DHEA; unlike DHEA, it is non-androgenic, non-anabolic, and an effective modulator of immune dysregulation. BEA is also an anti-infective agent and has been shown to benefit mycobacterial infections, including tuberculosis and leprosy. With the immune stabilizing capacity of BEA as well as its anti-mycobacterial properties, there is reason to believe that a randomized clinical trial with BEA may be beneficial for SPS.

## 1. Introduction

Stiff person syndrome (SPS) is a rare central nervous system disorder characterized by rigidity and painful muscle spasms in the axial and proximal limb muscles, often triggered by external stimuli. The stiffness primarily affects the truncal muscles, provoking spasms that result in postural deformities. Chronic pain, impaired mobility, and lumbar hyperlordosis are common symptoms [1]. SPS was first identified in 1956 by Moersch and Woltman, who described a series of 14 patients experiencing progressive, fluctuating tightness in the spinal, abdominal, and thigh muscles [2]. SPS is estimated to have a prevalence of about one per million people. It affects women up to three times as frequently as men, making the condition originally called “stiff-man” syndrome a misnomer [3].

Some reports suggest that SPS is under-recognized [4,5,6], and a recent study from Colorado would bear that out as they report an SPS prevalence of 2.11 per 100,000 persons [7], more than 20-fold the typically reported rate of one per million [8,9].

While there are three classifications of SPS: classic, partial variants, and progressive encephalomyelitis with rigidity and myoclonus (PERM), most with SPS have the classic form of the disease [10].

In SPS, muscle rigidity gradually progresses from the trunk to the limbs, impacting muscles closest to the trunk before spreading further. This stiffness in the limbs can disrupt balance and gait, leading to awkward, “statue-like” falls in which the individual is unable to extend their arms to break the impact. In addition to increasing stiffness, many individuals with SPS experience episodes of muscle spasms triggered by sudden movements or emotional stress, such as feeling startled or upset [11].

SPS is recognized as an autoimmune disease with the predominant antibodies against the enzyme glutamic acid decarboxylase (GAD) [12]. Anti-GAD antibodies are integral to the diagnosis and understanding of stiff person syndrome. GAD is the enzyme widely expressed within the central nervous system that catalyzes the conversion of the *excitatory* neurotransmitter l-glutamate to the *inhibitory* gamma-aminobutyric acid (GABA) [13,14].

## 2. GAD

Antibodies against GAD, originally linked to SPS, are now recognized to underlie a broader range of neurological conditions collectively referred to as “GAD antibody-spectrum disorders” (GAD-SD). This spectrum includes SPS, autoimmune epilepsy, limbic encephalitis, cerebellar ataxia, and nystagmus. These disorders share overlapping clinical features, highlighting their classification as autoimmune disorders of heightened neuronal excitability [15,16].

GAD exists in two isoforms, GAD65 and GAD67, that share a similar structure consisting of an amino-terminal domain, a catalytic domain binding the cofactor pyridoxal 5′-phosphate (PLP), and a carboxy-terminal domain [17]. Despite a common structure, GAD65 and GAD67 differ with regard to several characteristics, including their amino acid sequence [17], their molecular weight, their localization within the cell, and their enzymatic activity [18].

GAD65 is primarily expressed in the pre-synaptic end of nerve terminals where it exists in its inactive form; by switching from the inactive to the active form, GAD65 allows a rapid synthesis of inhibitory GABA when needed [18,19]. Antibodies against GAD reduce the availability of the neuroinhibitory GABA; SPS unchecked excitatory symptoms are due to impaired regulations of enzymatic conversion.

## 3. Anti-GAD in SPS and Autoimmune Diabetes (T1D)

Anti-GAD antibodies were first linked to SPS and later associated with the aforementioned spectrum of anti-GAD neurologic diseases: autoimmune epilepsy, limbic encephalitis, cerebellar ataxia, and nystagmus [15]. In a pivotal 1988 NEJM article, Solimena presented a case report of an individual with three anti-GAD disorders: SPS, autoimmune epilepsy, and autoimmune diabetes (T1D) [14]. Realizing that GAD is important both in the central nervous system and in pancreatic beta cells, the coexistence of SPS and diabetes has been broadly investigated [20,21]. Over 40% of individuals diagnosed with SPS also have T1DM [22]. Anti-GAD antibodies in SPS and T1D differ in both epitope specificity and the magnitude of antibody titers that in SPS may be 100-fold higher than in T1D [23,24].

The neuroendocrine functions of pancreatic beta cells, particularly their GABA-related activity mediated by GAD, highlight a complex regulatory system that parallels neuronal communication, emphasizing the intricate interplay between endocrine and nervous system mechanisms within the pancreas [14,25].

The identification and understanding of anti-GAD antibodies have significantly advanced the field of autoimmune diabetes research. A pivotal discovery occurred in 1990 when the 64 kDa islet cell antigen was identified as GAD. This enzyme, responsible for converting pancreatic glutamate to GABA, became a focal point in understanding autoimmune diabetes [26]. Research in the mid-1990s established that the presence of GAD autoantibodies could predict the development of type 1 diabetes prior to clinical symptoms. This discovery was crucial for early T1D diagnosis and intervention strategies [27].

T1D arises following the autoimmune destruction of the insulin-producing beta cells of the pancreas [28] and has been increasing globally starting after World War II [29]. Molecular mimicry has been suggested as a T1D mechanism where a foreign antigen provokes an immune response by cross-reacting with a similar-looking protein on one’s own pancreas (GAD) [30]. In searching for the protein involved in the mimicry, in 1982, a pancreatic protein was identified [31] that, in 1990, was strongly associated with a certain mycobacterial protein [32]. In one study, all newly diagnosed type 1 diabetic children were found to have immune responses to this mycobacterial protein named mycobacterial heat shock protein 65 (HSP65) [33].

## 4. GAD, Molecular Mimicry, *M. Paratuberculosis* and Mycobacterial HSP65

While mycobacterial diseases such as tuberculosis and leprosy have decreased in the developed world, another mycobacterium is on the rise: *Mycobacterium avium* subspecies *paratuberculosis* (MAP) [34,35,36]. MAP is historically well known as the infectious cause of fatal enteritis of ruminant animals, called Johne’s disease or paratuberculosis [37]. Humans are exposed to MAP in dairy products and meat [38,39,40,41].

Multiple studies suggest that exposure to cow’s milk is an “important determinant of subsequent type 1 diabetes in childhood” [42]. In 2006, Dow suggested that the immunogen triggering anti-GAD antibodies and, subsequently, T1D came from mycobacterial HSP65 and that the HSP65 was due to food contaminated by MAP [43].

Several follow-up studies were assessed in a systematic meta-analysis of the association between MAP and autoimmune diabetes that concluded: “… the study manifests a positive association between MAP and T1DM, highlighting that MAP prevention and environmental control would indubitably revolutionize T1DM management” [44].

HSP65 is an immunodominant protein exemplified by the fact that in human mycobacterial infection, HSP65 generates up to 40% of the T-cell response directed against this single protein [45]; moreover, mycobacterial HSP65 has been implicated in other human autoimmune diseases [46,47,48].

Anti-GAD65 antibodies in stiff-person syndrome (SPS) predominantly target linear epitopes, as evidenced by their recognition of GAD65 in Western blot analyses [49,50]. In contrast, anti-GAD antibodies of T1D recognize conformational similarities, indicating distinct antigenic specificity between these conditions [51,52].

## 5. SPS: Current Treatments

Antispasmodic agents are the first-line agents in SPS therapy; however, the amount and frequency of drugs required to address SPS symptoms commonly result in over-sedation. Therapeutic strategies based on the SPS pathophysiology target both the impaired reciprocal GABA inhibition to improve the primary clinical manifestations of stiffness in the truncal and proximal limb muscles, gait dysfunction, and episodic painful muscle spasms. SPS patients frequently have symptom progression requiring intravenous immunoglobulin (IVIg) [53,54].

Reviewing current and potential future treatment for SPS, Dalakas proposed the use of an interleukin-6 (IL-6) antagonist, a treatment already approved for neuromyelitis optica spectrum disorder (NMO-SD) [53]. This agent, satralizumab, is a subcutaneously administered, humanized monoclonal antibody that binds to both membrane-bound and soluble IL-6 receptors, preventing IL-6 from binding and inhibiting the IL-6 signaling pathways involved in inflammation [55,56]. The association of NMO-AD and MAP antigens [57,58] is of interest.

## 6. 16 Alpha-Bromoepiandrosterone—BEA

Dehydroepiandrosterone (DHEA) and its sulfate ester (DHEA-S) are the most abundant steroids found in the human metabolome, playing key roles in metabolism, immune regulation, and stress adaptation [59]. DHEA levels peak in early adulthood and decline with age, with levels of DHEA-S in older adults (ages 70–80) dropping to just 10–20% of those observed in younger individuals [60].

DHEA and cortisol exhibit opposing hormonal effects, and a proper balance between them is essential for various physiological functions [40]. While DHEA is often called the “youth hormone” [61,62,63], cortisol is widely known as the “stress hormone” [64]. A reduced DHEA-to-cortisol ratio has been associated with stress-related conditions, metabolic syndrome, immune system dysfunction, increased vulnerability to infections, frailty, and higher overall mortality [65,66,67,68,69]. Although the adrenal glands are the primary producers of DHEA and cortisol, these hormones are also synthesized locally in other tissues such as the skin, brain, heart, intestines, gonads, and lymphoid organs [65]. The decline in the DHEA/cortisol ratio with aging is linked to immune system changes characteristic of aging (immunosenescence) [70,71,72] and a paradoxical increase in inflammatory activity (inflammaging) [73].

16 alpha-Bromoepiandrosterone (BEA) is a synthetic derivative of DHEA that lacks the androgenic and estrogenic properties of the parent compound. BEA acts as a potent inhibitor of mammalian glucose-6-phosphate dehydrogenase (G6PDH), with approximately 60-fold greater potency than DHEA [74,75]. Developed over two decades ago for infectious disease treatment, BEA, also known as HE2000, has undergone nine clinical trials involving 228 participants for conditions such as HIV, malaria, and hepatitis [74,76,77,78]. In 1999, the FDA granted investigational new drug (IND) status to BEA for its potential use against HIV/AIDS, where it showed efficacy [79,80,81].

BEA has demonstrated the ability to modulate excessive inflammation [82] and, due to its broad immunomodulatory effects, is being explored as a therapeutic option for *Mycobacterium tuberculosis*, the world’s most significant pathogen [83,84].

As with DHEA, BEA promotes a T1 immune response and helps restore the Th1/Th2 balance, which tends to shift with age. Correcting this imbalance addresses age-related vulnerabilities, such as diminished vaccine efficacy, increased infection susceptibility, and higher rates of autoimmune disorders [85,86,87].

In earlier clinical trials, BEA was predominantly administered intramuscularly (IM) using an oil-based formulation, which led to mild to moderate injection site reactions, such as pain and induration [76,80]. The formulation has since been updated to a water-soluble version, which is expected to reduce or eliminate these adverse effects [88].

## 7. Discussion

The immunologic connection between SPS and T1D [89,90] is intriguing and has prompted this article’s proposal that MAP has a causal role in SPS as it has in T1D [44,48]. The range of treatment options for SPS commonly progresses to IVIg infusion; while IVIg remains an effective treatment for 67% of patients, in 30% of those, its efficacy decreases due to the natural progression of the disease [91]. Moreover, the cost of IVIg infusion ranges from USD 15,000 to USD 50,000 per month [92]. Clearly, alternative treatment options are needed for the SPS spectrum.

The relationship between MAP and SPS is speculative, and a clinical trial of BEA that targets immune dysregulation and mycobacterial infection would be required to support that relationship. Such a trial would include clinical outcome measures of stiffness index, spasm frequency, functional independence measurement, and pain monitoring. Objective measurement would include electromyography, GAD65 antibody titers, and gait analysis via motion sensors. Also, as BEA normalizes inflammatory cytokines IL-1b, IL-6, and TNF-a, measuring the change in these cytokines would enhance these clinical trials [93].

Conceptually, this would be a seamless Phase Ib/IIa trial, with Phase Ib focusing on safety, tolerability, and dose escalation of BEA and establishment of the recommended Phase II dose. The IIa would assess efficacy and continued safety monitoring in an effort to compare BEA to the current IVIg treatment.

Assessment of MAP infection status could be incorporated into the BEA/SPS trial with pretreatment and posttreatment blood sampling [94].

Dalakas, a thought leader in the diagnosis and management of SPS, states that while SPS is clearly an autoimmune disease, its management is hindered by obscure etiopathology wherein immune antigens have yet to be identified [91]. This article proposes that MAP provides the immunogen HSP65; further, the article proposes the treatment of both MAP and the inflammatory manifestations of SPS with BEA, a therapeutic agent that can treat inflammation and mycobacterial infection [83,84].

## Data Availability

No new data were created or analyzed in this study.
